# Supplementary material for: Antioxidants and Phenolic Acid Composition of Wholemeal and Refined-Flour, and Related Biscuits in Old and Modern Cultivars Belonging to Three Cereal Species
Source: Foods. 2023 Jun 29;12(13):2551. doi: 10.3390/foods12132551 (PMC10340153; doi:10.3390/foods12132551)

Figure S1. Example of phenolic and flavonoid chromatogram during the whole cereal food supply chain

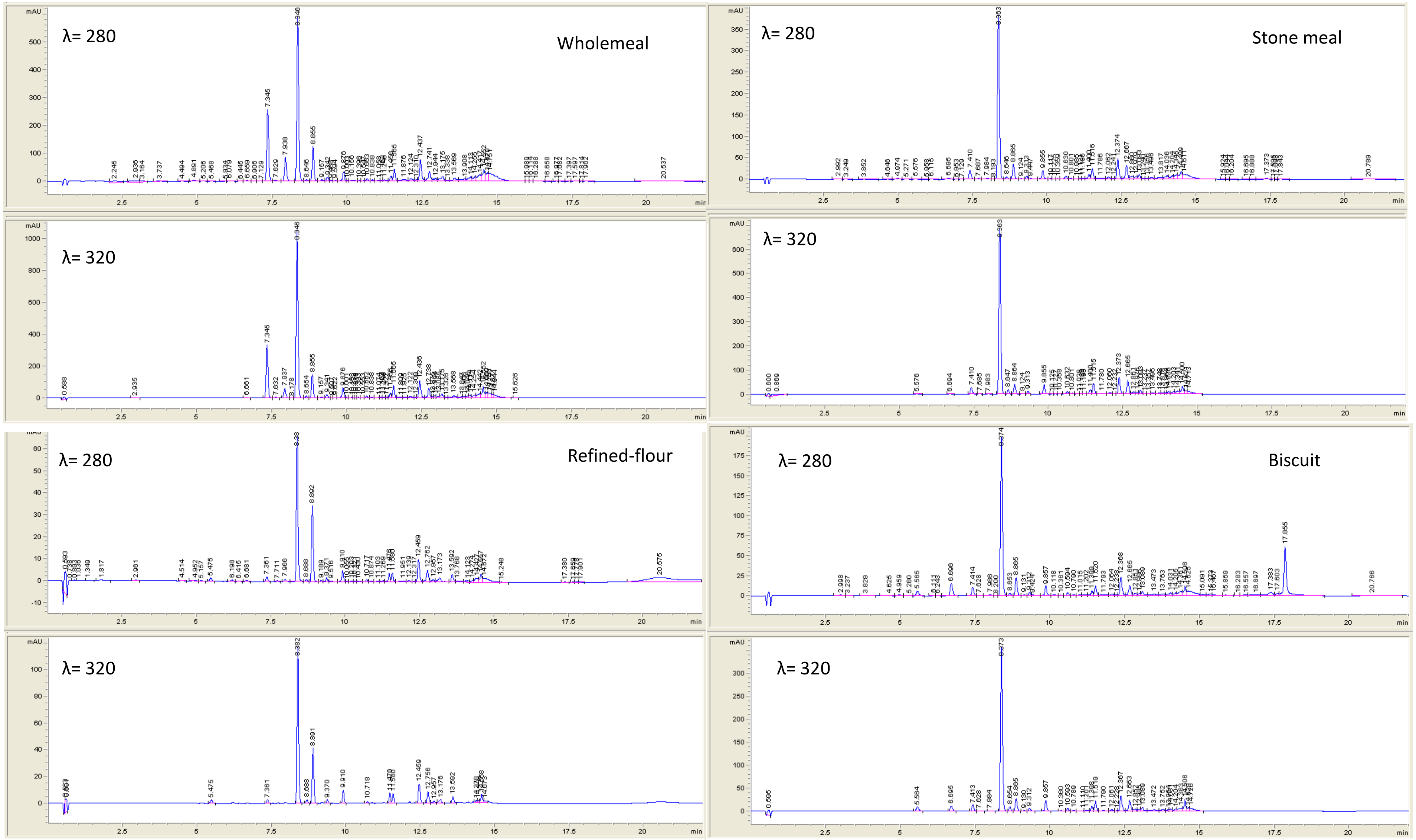

Supplement: Supplementary file 1 [file foods-12-02551-s001.zip › Figure S1_Borrelli et al_R1.pdf]
