# Supplementary material for: Antioxidants and Phenolic Acid Composition of Wholemeal and Refined-Flour, and Related Biscuits in Old and Modern Cultivars Belonging to Three Cereal Species
Source: Foods. 2023 Jun 29;12(13):2551. doi: 10.3390/foods12132551 (PMC10340153; doi:10.3390/foods12132551)
Supplement: Supplementary file 1 [file foods-12-02551-s001.zip › Supplementary Table S1_Borrelli et al_R1.pdf]

Table S1. Monthly total rainfall and temperature (minimum and maximum) at the experimental field of Foggia during the two growing seasons, 2016 and 2017

	**Rainfall (mm)**		**Min. temperature (°C)**		**Max. temperature (°C)**	
	**2016**	**2017**	**2016**	**2017**	**2016**	**2017**
Nov	57	38.4	6.8	7.3	17.2	17.1
Dec	0.4	3.1	3.3	1.7	13.9	13.1
Jan	15.4	81.8	3.3	1.1	13.1	8.2
Feb	24.4	23.2	4.5	5.3	16.3	14.8
Mar	114.8	8.2	5.1	5.2	14.6	17.6
Apr	32.2	23.5	9.2	5.5	21.5	18.9
May	72.9	80	10.4	10.5	23.2	25.0
Jun	58.4	2.6	15.5	17.1	28.9	32.6
Mean			*7.9*	*7.3*	*18.9*	*18.7*
Total	*375.5*	*260.8*				
