# Supplementary material for: Antioxidants and Phenolic Acid Composition of Wholemeal and Refined-Flour, and Related Biscuits in Old and Modern Cultivars Belonging to Three Cereal Species
Source: Foods. 2023 Jun 29;12(13):2551. doi: 10.3390/foods12132551 (PMC10340153; doi:10.3390/foods12132551)
Supplement: Supplementary file 1 [file foods-12-02551-s001.zip › Supplementary Table S5_Borrelli et al_R1.pdf]

Table S5. Eigenvalue and percentage of variation explained by the first eleven factors for all traits analyzed.

	Principal components										
	1	2	3	4	5	6	7	8	9	10	11
Eigenvalues	10.667	4.433	3.060	2.242	1.269	0.983	0.564	0.434	0.207	0.093	0.050
PC variation (%)	44.444	18.469	12.751	9.34	5.287	4.096	2.35	1.807	0.861	0.388	0.208
Cumulative variation (%)	44.444	62.913	75.664	85.004	90.291	94.387	96.737	98.544	99.405	99.793	100.001
**Trait**	*Unrotated factors*										
PC	-0.446	-0.411	0.205	0.622	-0.334	0.049	0.013	0.282	-0.085	-0.053	-0.012
TW	-0.185	0.691	0.566	0.217	-0.006	0.241	0.223	-0.094	-0.014	0.021	-0.057
TKW	-0.282	0.809	0.372	0.058	-0.220	0.050	0.193	-0.002	0.149	-0.114	0.037
TPC	0.969	-0.161	0.045	0.122	-0.013	0.063	-0.017	0.100	-0.003	-0.050	-0.024
TFC	0.969	-0.133	0.171	0.071	-0.031	0.018	0.004	0.074	-0.042	-0.005	0.013
TPAC	0.926	-0.098	0.297	0.088	-0.142	0.070	-0.007	-0.079	-0.020	0.078	-0.009
TYPC	-0.801	0.414	0.035	-0.089	0.213	-0.317	0.034	0.039	-0.149	0.069	-0.057
DPPH	0.964	-0.137	0.183	0.102	-0.057	0.060	-0.001	0.011	-0.029	-0.023	0.008
TEAC	0.972	-0.073	0.184	0.086	-0.065	0.052	0.000	-0.009	-0.032	0.023	-0.025
Protocatechuic acid	-0.648	-0.316	0.364	0.264	0.276	-0.090	0.427	0.071	-0.077	0.023	0.015
p -Hydroxybenzoic acid	-0.220	-0.519	0.400	0.278	0.539	0.373	-0.023	-0.060	0.093	0.031	0.033
Vanillic acid	0.430	0.528	-0.188	-0.573	-0.104	0.185	0.135	0.324	-0.018	-0.027	0.058
Caffeic acid	0.821	-0.102	-0.303	0.123	0.347	-0.143	0.092	-0.189	-0.018	-0.148	0.046
Syringic acid	0.785	-0.245	-0.208	-0.239	0.371	-0.095	0.256	0.075	-0.052	-0.043	-0.012
Vanillin	-0.060	0.598	0.227	-0.245	0.543	0.309	-0.313	0.193	-0.025	-0.015	-0.021
p-Coumaric acid	0.870	-0.102	-0.309	-0.244	0.027	-0.208	0.087	0.025	0.119	0.046	-0.097

Syringaldeide	0.047	0.128	-0.736	0.539	0.014	0.354	0.039	-0.051	0.116	-0.018	-0.073
Ferulic acid	0.662	0.524	-0.253	0.402	0.025	-0.013	-0.073	-0.119	-0.152	0.086	0.105
Sinapic acid	-0.878	-0.336	-0.115	-0.233	-0.150	-0.002	-0.080	-0.109	0.075	-0.009	0.054
trans-Cinnamic acid	0.539	0.096	0.739	-0.273	0.027	-0.180	0.047	-0.067	0.187	0.062	0.024
cis-Cinnamic acid	0.152	0.616	-0.615	0.364	0.086	-0.044	0.146	0.145	0.132	0.122	0.047
Narigenin	-0.175	-0.945	-0.086	-0.017	0.047	0.018	0.018	0.220	0.105	0.061	0.040
Catechin	0.900	-0.091	0.350	0.061	-0.195	0.117	-0.035	0.033	-0.031	0.039	-0.001
Quercitin	-0.188	-0.319	-0.297	-0.606	-0.179	0.529	0.239	-0.159	-0.107	0.049	0.001
